# 

**Published:** 1872-03

**Authors:** 


### INDEX OF SUBJECTS.

### ORIGINAL COMMUNICATIONS.

Causes and Treatment of Obesity,.. ........................................ ..129
Necrosis, with Spontaneous Resection of Lower Maxilla,....................134
Chronic Otorrhœa,........................................................136
Remarks on Neuralgia,... ................................................138
Reports of Some Interesting Cases from the Surgical Clinics and Hospital of Dr. Van Langenbeck, of Berlin, Prussia.......................................143

### EXTRACTS AND GLEANINGS.

Addison's Disease - Furunculous Abscess in the External Meatus—Croton Chloral Iron in Uterine Injections, 150; Management of Epilepsy—Strychnia in Hypodermic, 151; The Cure of Cancer by Electrolysis—Carded Wool in the Treatment of Wounds, 152; Treatment of Bubo—Arsenic in Menorrhagia and Leucorrhœa, 153; Treatment of Diptheria with Carbolic Acid—Taraxacum Coffee, 154; Cardialgia (Heartburn)—Apiol, 155; Uterine Tonic—The Relations of Asthma, Angina Pectoris, and Gastralgia, 156; Strychnia in Albuminuria—Dr. Debout's Pills for Migraine—Chloral for Toothache—Ioduretted Gargle for Syphalitic Ulceration of the Mouth—Bulimic Dyspepsia, 157; Collodium-Paper—Chorea Treated by Sulphate of Zinc—Glandular Enlargements, 158; Chloral—Anti Neuralgic Powders—Treatment of Small Pox, 159; Bromide of Calcium—Vomiting of Pregnancy, 160; Cod-Liver Oil—Pepsine—Pancreatine—Carminative Powder for Infants, 161; Syphilis—Eulenberg's Formula for Vomiting of Pregnancy, 162; Fistula in Ano—Fever Mixture—Liebreich's Operation for Extraction of Cataract, 163; Partial Hemiplegia—Biliary Calculi, 164; Vaginismus—Tetanus, 165; Observations on Rachitis—How to Prevent the Rusting of Steel Instruments, 165; Drops for Gastralgia—Foot and Mouth Disease in Children—Carbolized Gargle for Diptheria—Tincture of Capsicum as a Styptic, 167; Ulcers of the Leg—A Remedy for Freckles, 168; Deficient Secretion—Anæsthetic Calcareo-Glycerine for Burns—Strychnia in Albuminuria, 169; Gallic Acid in Uterine Hemorrhage—Cure for Toothache—Drastic Pills for Dropsy—Popliteal Aneurism Cured by Flexion in Three Days, 170; Ipecacuanha Given in Enema for Dysentery, 171; Sulphate of Iron as a Local Application in Phlegmasia Dolens—Catarrhus Vesicæ—Carbonate of Ammonia in Pneumonitis, 172; Cure of Hydrocele by the Seton—Van Den Court's Pills for Chordee—The Teaspoon as a Measure, 173; Subcutaneous Injections of Ergotin in Uterine Affections, 174; Therapeutical Use of Water—Carminative Mixture—Uva Ursi as an Emmenagogue—Cystalgia, 175; Ether Spray in Diagnosis—Formula for Cystitis, 176.

### EDITORIALS, REVIEWS, ETC.

Friends, Take Notice, and Act,...........................................177
Our Thanks—Please Remember—American Medical Association,...............178
A Splendid Number—Success of the Companion,.............................179
Literary Periodicals—Dr. Geo. C. Crawford—National Medical Journal,........180
The Truth Forcibly Spoken,...............................................181
Books and Pamphlets,.....................................................188
Extracts from our Correspondents,........................................191

